# Lung ultrasound radiomics for identifying severe *Mycoplasma pneumoniae* pneumonia in children: a model development study

**DOI:** 10.3389/fped.2026.1927948

**Published:** 2026-09-22

**Authors:** Pengcheng Zhang, Rong Wu, Wenjing Liu, Peng Hao, Yang Xin, Huiwen Li

**Affiliations:** 1 Ordos Clinical Medical College, Inner Mongolia Medical University, Ordos, China; 2 Department of Ultrasound, Ordos Central Hospital, Ordos, China

**Keywords:** children, lung ultrasound, machine learning, *Mycoplasma pneumoniae* pneumonia, radiomics, random forest, severe pneumonia

## Abstract

**Background:**

*Mycoplasma pneumoniae* pneumonia (MPP) is a common cause of community-acquired pneumonia in children, but objective identification of severe MPP (SMPP) remains challenging. This study aimed to develop and internally validate lung ultrasound radiomics–clinical models for distinguishing SMPP from non-severe MPP (NSMPP).

**Methods:**

This retrospective study included children younger than 14 years with MPP between July 2020 and June 2025. Participants were allocated by stratified random sampling to training and held-out internal testing cohorts at an 8:2 ratio. Clinical data were collected within 24 h after admission, and lung ultrasound was performed within 72 h. Radiomics features were extracted from manually segmented pulmonary consolidations using PyRadiomics and selected by interobserver reproducibility assessment, correlation analysis, and least absolute shrinkage and selection operator regression. Clinical, Radiomics, and Integrated random forest models were developed, with hyperparameters optimized in the training cohort using stratified five-fold cross-validation. Model discrimination, calibration, clinical utility, and interpretability were evaluated using receiver operating characteristic analysis, calibration curves, decision curve analysis, pairwise comparisons, and SHapley Additive exPlanations (SHAP).

**Results:**

Overall, 530 children were included: 424 in the training cohort and 106 in the testing cohort. Training AUCs for the Clinical, Radiomics, and Integrated models were 0.785 (95% CI, 0.740–0.826), 0.794 (95% CI, 0.748–0.836), and 0.823 (95% CI, 0.780–0.863), respectively. Corresponding testing AUCs were 0.747 (95% CI, 0.646–0.837), 0.787 (95% CI, 0.683–0.877), and 0.801 (95% CI, 0.699–0.889). Although the Integrated model achieved the highest testing AUC, its improvement over the Radiomics model was small (*Δ*AUC = 0.014) and not significant by the DeLong test (*P* = 0.246) or integrated discrimination improvement analysis (*P* = 0.288). SHAP identified transformed grayscale intensity, texture organization, lesion morphology, and lactate dehydrogenase as important predictors.

**Conclusions:**

The Integrated model showed the highest discrimination, but a definite incremental benefit over the Radiomics model was not demonstrated. Lung ultrasound radiomics may provide a non-invasive quantitative approach for identifying SMPP in children; however, prospective multicenter external validation is required before clinical implementation.

## Introduction

*Mycoplasma pneumoniae* pneumonia (MPP) is one of the most common etiologies of pediatric community-acquired pneumonia (CAP), accounting for 10%–40% of hospitalized CAP cases in children (1–3). In recent years, following the global resurgence of *Mycoplasma pneumoniae* (MP) infections in the post-COVID-19 era, the incidence of MPP has increased substantially, while the widespread emergence of macrolide-resistant strains has further complicated clinical management (4, 5). Approximately 10%–30% of children with MPP may progress to severe *Mycoplasma pneumoniae* pneumonia (SMPP) or refractory *Mycoplasma pneumoniae* pneumonia (RMPP), characterized by persistent high fever, extensive pulmonary consolidation, massive pleural effusion, pulmonary necrosis, and even severe complications such as plastic bronchitis. Some affected children require bronchoscopic intervention, glucocorticoid therapy, and second-line antibiotics, resulting in significantly prolonged hospitalization; in the long term, sequelae such as bronchiolitis obliterans may occur (6, 7). Therefore, early identification and quantitative assessment of the risk of progression to severe disease in children with MPP are of considerable clinical importance for guiding risk-stratified treatment, optimizing healthcare resource allocation, and improving patient outcomes.

Lung ultrasound (LUS) has gained increasing attention in recent years as a bedside imaging modality for the assessment of pediatric pneumonia (8). Compared with chest radiography and computed tomography (CT), LUS offers several advantages, including the absence of ionizing radiation, feasibility for bedside use, high repeatability, and low cost, and has demonstrated unique value in the early diagnosis, disease monitoring, and evaluation of complications in children with pneumonia (9). By semi-quantitatively assessing sonographic features such as disappearance of A-lines, the number of B-lines, the extent of pulmonary consolidation, and pleural effusion, LUS-based scoring systems can provide an objective reflection of the severity of pulmonary lesions (10, 11). Xie et al. (12) applied a modified lung ultrasound score (MLUS) (13) for etiological differentiation in children with MPP and found that the MLUS was significantly higher in the MP infection group (with a median score of 15) than in the *bacterial* pneumonia group (median score: 9) and viral pneumonia group (median scores 8). These findings suggest that LUS may provide supplementary imaging information for assessing pulmonary involvement and disease severity when interpreted together with clinical and laboratory findings.

Ultrasound radiomics, an emerging branch of radiomics, has made substantial progress in recent years in the differential diagnosis and prognostic evaluation of tumors involving superficial and abdominal organs, such as the thyroid, breast, and liver. However, its application in pulmonary diseases remains at an early stage (14, 15). Introducing radiomics-based ultrasound approaches into the field of MPP is expected to enable the quantitative characterization of microscopic heterogeneity in pulmonary parenchymal lesions under radiation-free conditions (16). By integrating clinical features to construct multidimensional predictive models, this approach may provide a novel technical pathway for the early identification of SMPP. At present, no reports have been published domestically or internationally on the application of lung ultrasound radiomics in MPP. This research gap not only reflects the challenges of technological translation.

This study aims to develop an ultrasound-based Radiomics model for identifying children at risk of developing severe *Mycoplasma pneumoniae* pneumonia (SMPP). By integrating radiomic features with clinical data, we seek to establish a robust predictive model that can support early risk stratification and individualized clinical decision-making.

## Materials and method

### Patient cohorts

This study follows the Declaration of Helsinki and was approved by the Ethics Committee of the Ordos Central Hospital (grant no. 2026-147) and also waived informed consent. A retrospective case collection was conducted at the Ordos Central Hospital from July 2020 to June 2025. The enrolled patients were first classified as having non-severe *Mycoplasma pneumoniae* pneumonia (NSMPP) or SMPP according to the predefined national diagnostic criteria (17). Subsequently, the entire cohort was randomly divided into a training cohort and a testing cohort at a ratio of 8:2 using stratified random sampling based on disease severity. The training cohort was used for feature selection, model development, and hyperparameter optimization, whereas the independent testing cohort was reserved exclusively for internal validation of model performance.

The inclusion criteria were as follows: (1) diagnosis of MPP in patients under 14 years of age according to national diagnostic criteria (17); (2) availability of complete clinical and laboratory data; and (3) availability of lung ultrasound examination performed within 72 h after admission.

The exclusion criteria were as follows: (1) pneumonia caused by other definite pathogens, including bacteria, viruses, fungi, or tuberculosis; (2) mixed infection in which the predominant role of MPP could not be clearly determined; (3) pre-existing chronic pulmonary diseases, congenital heart disease, immunodeficiency, malignancy, or severe hepatic, renal, or hematological disorders; (4) history of thoracic surgery, chest trauma, or other conditions that could substantially affect lung ultrasound findings; (5) incomplete clinical, laboratory, or imaging data; and (6) poor-quality lung ultrasound images that were unsuitable for lesion assessment or radiomics feature extraction.

Based on the national diagnostic criteria (17), patients were classified as having SMPP if they met at least one of the following criteria: (1) persistent high fever, defined as a body temperature of ≥39.0 °C for ≥5 days, or fever lasting ≥7 days without a decrease in peak temperature or with a further increase; (2) wheezing, tachypnea, dyspnea, chest pain, or hemoptysis; (3) extrapulmonary organ involvement that did not meet the criteria for critical illness; (4) resting peripheral oxygen saturation of ≤93% while breathing room air; (5) chest radiographic or CT findings showing high-density consolidation involving two-thirds or more of a single pulmonary lobe, consolidation involving at least two pulmonary lobes regardless of the extent within each lobe, or unilateral or bilateral diffuse bronchiolitis, with or without bronchial wall thickening, peribronchial infiltration, mucus plugging, or atelectasis; (6) clinical deterioration accompanied by an increase of >50% in the estimated extent of pulmonary lesions relative to baseline on repeated chest radiography or CT performed within 24–48 h; or (7) a marked elevation in at least one of the following laboratory markers: C-reactive protein, lactate dehydrogenase, or D-dimer. Patients who met none of these criteria were classified as having NSMPP.

The imaging criteria were assessed using chest radiography or CT and were independent of the lung ultrasound images used for radiomics analysis. Two radiologists independently reviewed the chest images and visually estimated the extent of lobar involvement by comparing the affected area with the total area of the corresponding lobe. Disagreements were resolved by consensus. All included patients underwent lung ultrasound within 72 h after admission. Accordingly, “diffuse bronchiolitis” referred to findings on chest radiography or CT and did not refer to confluent B-lines on lung ultrasound. A schematic overview is shown in Figure 1.

**Figure 1:**
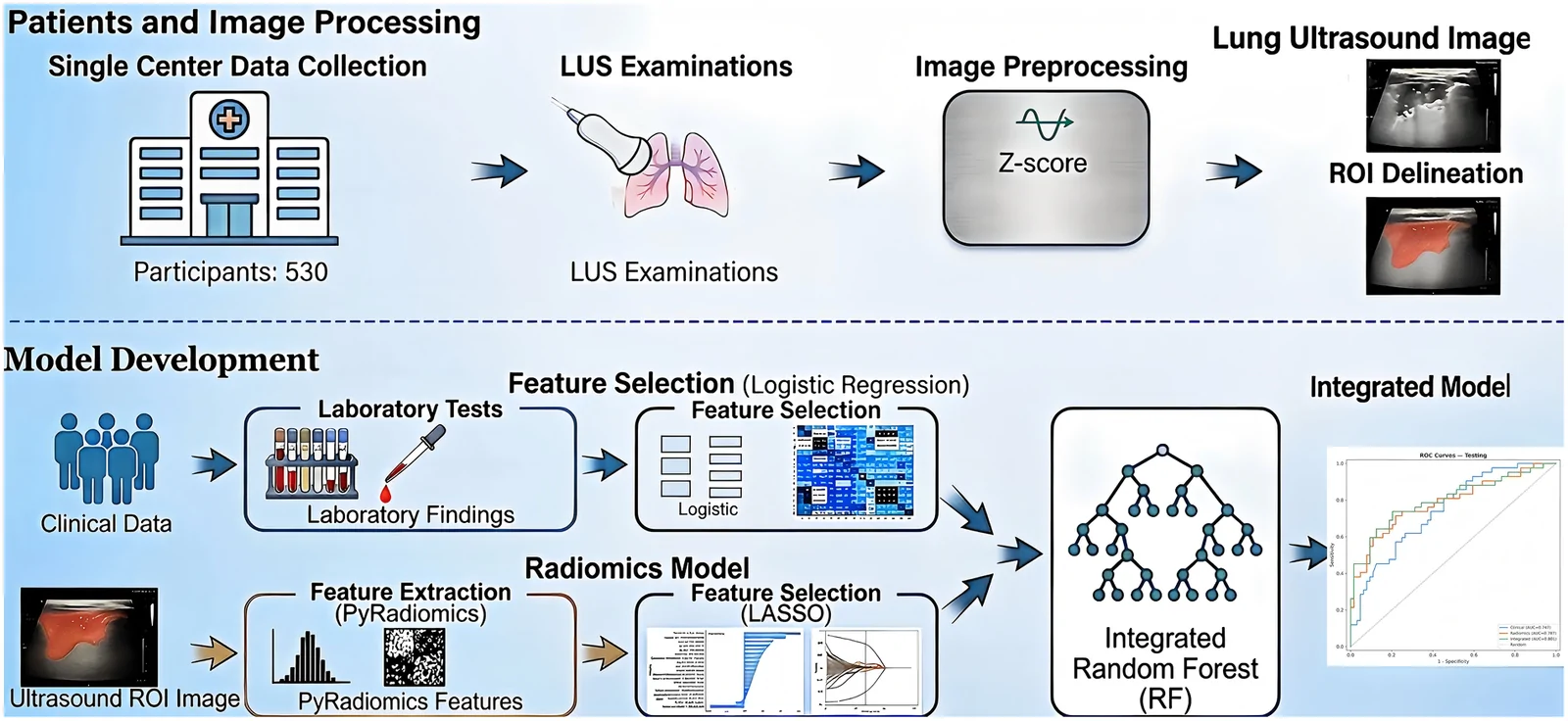
Flow chart of the study. LUS, Lung Ultrasound; ROI, Region of Interest; LASSO, Least Absolute Shrinkage and Selection Operator.

### Data sets

Patient data were retrospectively collected and included demographic information, laboratory findings, and imaging characteristics. Demographic variables included sex and age. Laboratory data were obtained from the first blood test performed within 24 h after admission. The laboratory parameters included white blood cell (WBC) count, absolute neutrophil count (NEUT_value), neutrophil percentage (NEUT%), absolute lymphocyte count (LYMPH_value), lymphocyte percentage (LYMPH%), monocyte count (MONO), platelet count (PLT), C-reactive protein (CRP), procalcitonin (PCT), alanine aminotransferase (ALT), aspartate aminotransferase (AST), creatine kinase (CK), creatine kinase-MB isoenzyme (CK-MB) and lactate dehydrogenase (LDH). The systemic immune-inflammation index (SII) was calculated from laboratory measurements obtained at the same time point using the following formula: SII = platelet count ×  absolute neutrophil count/absolute lymphocyte count.

The examinations were performed using LOGIQ 7C, HITACHI HI VISION Ascendus, Canon Aplio i900, or Mindray Resona R7/R9/A20S ultrasound systems equipped with linear-array or convex-array transducers. Children were examined in the sitting or supine position according to age and clinical condition. An appropriate transducer was selected, and both lungs were scanned in longitudinal planes, perpendicular to the chest wall, and transverse planes, parallel to the intercostal spaces. Both lungs were systematically evaluated using a 12-zone protocol, with six regions on each side. Longitudinal and transverse scans were obtained for each region. Imaging depth, overall gain, focal-zone position, and dynamic range were adjusted to optimize lesion visualization. During the examination, the image section showing the largest consolidation area with the clearest image quality was selected and saved in PNG format to preserve as much original image information as possible. Image quality was independently assessed by two radiologists with at least 5 years of experience in pediatric lung ultrasound. Disagreements were resolved by consensus with a senior radiologist with 8 years of experience. The lung consolidation areas on all grayscale ultrasound images were manually segmented. Regions of interest were independently delineated by two sonographers who were blinded to the patients’ clinical information. Sonographer 1 had at least 5 years of experience in lung ultrasound, whereas Sonographer 2 had at least 2 years of experience. Both sonographers used ITK-SNAP software, version 3.8.0, to delineate the region-of-interest contours on the largest plane of each consolidation lesion. When the difference between the two segmentations exceeded 5% of the ROI area, the case was reviewed by a senior sonographer and resolved by consensus. To assess interobserver reproducibility, 50 cases were randomly selected and independently segmented by two sonographers who were blinded to each other's segmentation results. Radiomics features were separately extracted from the two sets of segmentations. Interobserver reproducibility was assessed using the intraclass correlation coefficient based on a two-way random-effects model with absolute agreement. Features with ICCs greater than 0.75 were considered reproducible and retained for subsequent analysis. The interobserver ICCs ranged from 0.823 to 0.999, indicating good-to-excellent interobserver reproducibility.

### Radiomics feature extraction and selection

The handcrafted features were divided into three groups: (I) geometry, (II) intensity, and (III) texture. Geometry features describe the two-dimensional shape characteristics characteristics of the lesion, while intensity features characterize the first-order statistical distribution of pixel-intensity distribution within the lesion. Texture features reflect the second- and higher-order spatial distributions of intensities and were extracted using several methods, including the gray-level co-occurrence matrix (GLCM), gray-level run-length matrix (GLRLM), gray-level size-zone matrix (GLSZM), and neighboring gray-tone difference matrix (NGTDM). Regions of interest were manually segmented on lung ultrasound images, and radiomics features were extracted from each segmented lesion using PyRadiomics version 3.0.1.

#### Statistics

We also conducted Mann–Whitney U test statistical test and feature screening for all radiomic features. Only the *P* value < 0.05 of radiomic feature were kept.

#### Correlation

For features with high repeatability, Spearman's rank correlation coefficient was also used to calculate the correlation between features, and one of the features with correlation coefficient greater than 0.9 between any two features is retained. In order to retain the ability to depict features to the greatest extent, we use greedy recursive deletion strategy for feature filtering, that is, the feature with the greatest redundancy in the current set is deleted each time.

#### Lasso

LASSO regression was applied to the radiomics features in the training cohort for feature selection. The regularization parameter *λ* was determined using 10-fold cross-validation based on the minimum cross-validation error criterion. Features with nonzero coefficients at the optimal *λ* were retained.

#### Model development

Three random forest models were developed: a Clinical model based on the selected clinical features, a Radiomics model based on the radiomics features retained after LASSO selection, and an Integrated model combining the selected clinical features with the retained radiomics features. For each model, hyperparameters were optimized separately and exclusively within the training cohort using grid search with stratified five-fold cross-validation, with the mean cross-validated ROC AUC as the optimization criterion. The same optimal hyperparameter combination was identified for all three models: 200 trees, a maximum tree depth of 3, a minimum of 30 samples per leaf, and a minimum of 30 samples required to split an internal node. Class imbalance was addressed using balanced class weights without synthetic oversampling. All feature-selection procedures were restricted to the training cohort, and the held-out testing cohort was not involved in feature selection or hyperparameter optimization, thereby reducing the risk of information leakage. After hyperparameter selection, each model was refitted using the entire training cohort and evaluated once in the held-out internal testing cohort.

### Statistical analysis

Continuous variables were assessed for normality. Normally distributed variables were presented as mean ± standard deviation (SD) and compared using the independent-samples Student's *t*-test, whereas non-normally distributed variables were presented as median (interquartile range [IQR]) and compared using the Mann–Whitney *U* test. Categorical variables were expressed as frequencies and percentages and compared using Pearson's *χ*^2^ test or Fisher's exact test, as appropriate.

For the selection of clinical variables, potential predictors were first evaluated using univariate logistic regression. Variables with a *P*-value < 0.05 were selected for collinearity assessment using Spearman correlation analysis and the variance inflation factor (VIF). After excluding variables with multicollinearity (VIF > 5), the remaining independent variables were entered into a multivariate logistic regression model to determine the final independent risk factors.

The predictive performance of the models in assessing the severity of MPP was evaluated using the Receiver Operating Characteristic curves (ROC) and area under the ROC (AUC) with corresponding 95% confidence intervals. Additional performance metrics including sensitivity, specificity, accuracy, F1 score and precision were calculated to provide a comprehensive assessment of model effectiveness. Pairwise comparisons of model performance were conducted using the DeLong test and integrated discrimination improvement (IDI) analysis. To enhance interpretability of the Integrated model, we performed SHAP (SHapley Additive Explanations) analysis to evaluate the relative contribution of each feature to the model's output.

All statistical analyses were performed using R software (version 3.6.0; The R Foundation for Statistical Computing). A two-sided *p*-value < 0.05 was considered statistically significant.

## Results

### Patient characteristics

To ensure that the ultrasound findings reflected the early clinical status rather than subsequent disease progression or treatment effects, only patients who underwent lung ultrasound within 72 h after admission were eligible. The numbers of patients excluded for each reason, including unavailable ultrasound examinations, examinations performed beyond the predefined 72-h window, incomplete clinical data, and inadequate image quality, are presented separately in the flowchart. A total of 424 patients were included in the training cohort, while 106 patients comprised the testing cohort. The detailed inclusion and exclusion process is depicted in Figure 2. Extrapulmonary manifestations were documented in 268 of the 530 patients (50.6%). At least one extrapulmonary manifestation was recorded in 72 of 322 patients with NSMPP (22.4%) and 196 of 208 patients with SMPP (94.2%). The prevalence of extrapulmonary manifestations was significantly higher in the SMPP group than in the NSMPP group (94.2% vs. 22.4%; *χ*^2^ = 261.132, *P* < 0.001). As detailed in Supplementary Table S1.

**Figure 2:**
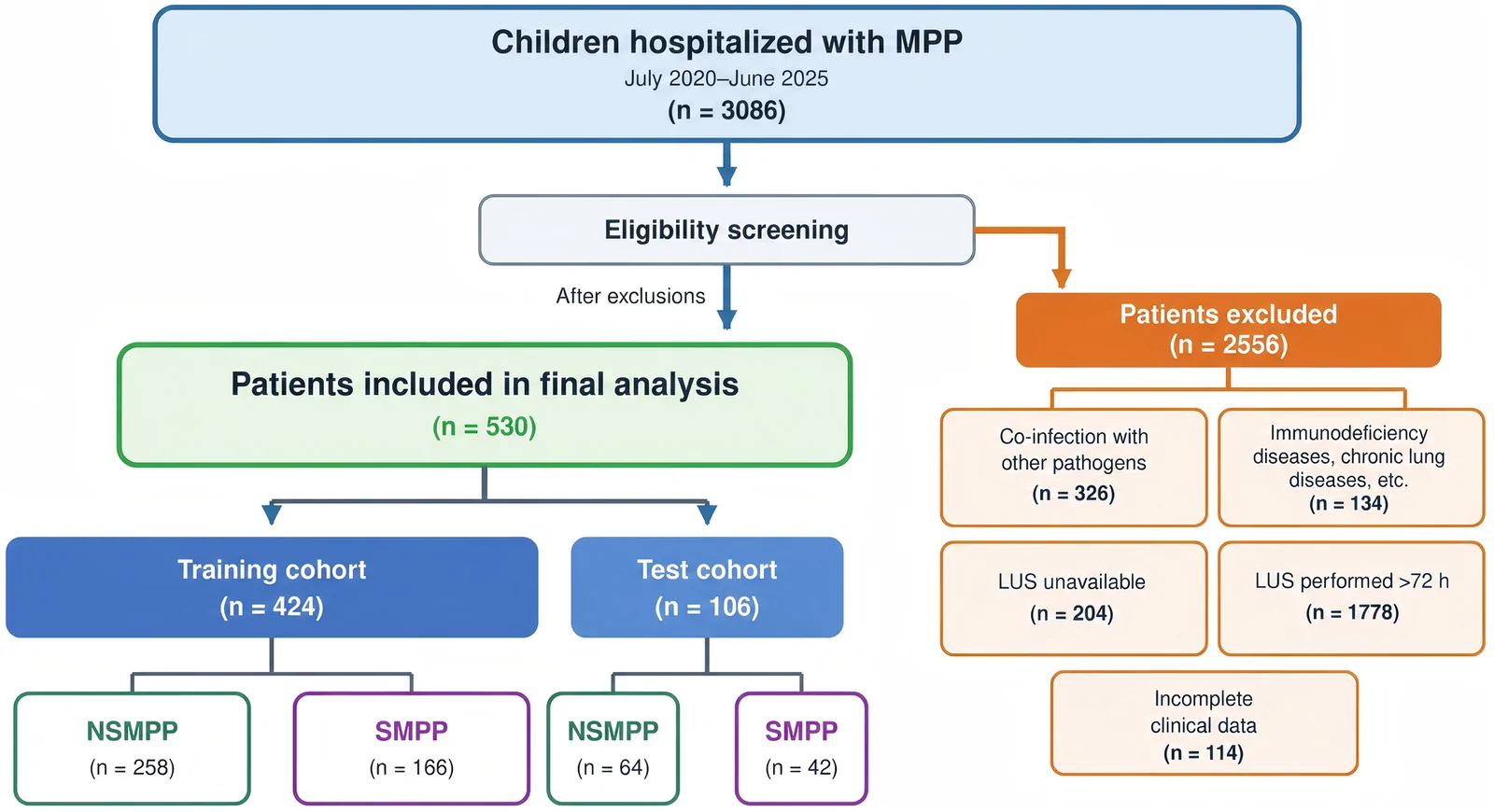
Flowchart of participant selection: adherence to inclusion and exclusion criteria in a single-center study. NSMPP, Non-Severe *Mycoplasma pneumoniae* Pneumonia; SMPP, Severe *Mycoplasma pneumoniae* Pneumonia.

Clinical characteristics according to disease severity in the training and testing cohorts are summarized in Table 1. In the training cohort, patients with SMPP showed higher distributions of age, neutrophil percentage, procalcitonin, alanine aminotransferase, lactate dehydrogenase, and systemic immune-inflammation index, together with lower lymphocyte percentage, lymphocyte count, monocyte count, and CK-MB levels than patients with NSMPP (all *P* < 0.05). In the testing cohort, patients with SMPP had lower lymphocyte counts and higher procalcitonin, aspartate aminotransferase, and lactate dehydrogenase levels (all *P* < 0.05). Among the evaluated variables, lymphocyte count, procalcitonin, and lactate dehydrogenase differed significantly between the NSMPP and SMPP groups in both cohorts. No statistically significant differences in the overall distributions of the listed clinical characteristics were observed between the training and testing cohorts (all *P* > 0.05) (Table 1).

**Table 1:** Comparison of clinical and radiological characteristics between NSMPP and SMPP groups in training and testing cohorts.

Feature	Training cohort(*n* = 424)		*P*-Intra value	Testing cohort(*n* = 106)		*P*-Intra value	Overall (*n* = 530)
	NSMPP (*n* = 258)	SMPP (*n* = 166)		NSMPP (*n* = 64)	SMPP (*n* = 42)		*P*-inter value
Age (years)	7.0 [4.0, 8.0]	7.0 [5.0, 9.0]	0.007	7.0 [4.8, 8.0]	7.0 [5.0, 9.0]	0.194	0.768
WBC (×10^9^/L)	7.3 [5.6, 9.3]	6.6 [5.6, 8.9]	0.253	7.1 [5.7, 8.3]	6.7 [5.8, 9.3]	0.799	0.528
NEUT (%)	64.1 [55.6, 69.6]	65.3 [56.8, 72.6]	0.011	63.2 [53.6, 67.0]	64.0 [53.8, 73.1]	0.257	0.261
NEUT_value (×10^9^/L)	4.6 [3.4, 5.9]	4.2 [3.5, 6.4]	0.874	4.3 [3.4, 5.4]	4.5 [3.4, 5.4]	0.572	0.366
LYMPH (%)	27.2 [21.6, 34.3]	23.8 [19.4, 32.2]	0.001	28.1 [22.6, 36.6]	24.9 [18.4, 35.2]	0.119	0.915
LYMPH_value (×10^9^/L)	1.9 [1.5, 2.6]	1.6 [1.1, 2.2]	<0.001	2.0 [1.4, 2.5]	1.5 [1.1, 2.4]	0.042	0.467
MONO (×10^9^/L)	0.52 [0.40, 0.67]	0.48 [0.35, 0.63]	0.013	0.54 [0.41, 0.71]	0.46 [0.36, 0.67]	0.234	0.699
PLT (×10^9^/L)	253.5 [215.8, 324.2]	249.5 [205.2, 303.8]	0.335	271.0 [228.0, 317.5]	244.5 [198.0, 298.2]	0.132	0.557
CRP (mg/L)	12.6 [4.8, 25.0]	11.7 [4.8, 26.2]	0.839	10.2 [5.8, 17.3]	10.9 [5.3, 26.7]	0.391	0.615
PCT (ng/mL)	0.12 [0.08, 0.19]	0.13 [0.08, 0.23]	0.040	0.11 [0.08, 0.15]	0.15 [0.09, 0.28]	0.013	0.871
ALT (U/L)	18.0 [15.0, 22.0]	20.0 [16.0, 26.0]	0.008	19.0 [16.0, 22.0]	20.0 [16.2, 25.8]	0.139	0.664
AST (U/L)	33.5 [29.0, 40.0]	36.0 [27.5, 41.0]	0.148	33.0 [29.0, 37.5]	37.5 [32.0, 44.8]	0.032	0.592
CK (U/L)	84.5 [64.8, 111.2]	83.5 [52.2, 142.5]	0.718	82.5 [57.8, 117.2]	89.0 [65.8, 135.0]	0.527	0.647
CK-MB (U/L)	21.8 [15.7, 32.8]	19.3 [15.5, 27.8]	0.047	20.6 [15.2, 24.5]	19.3 [15.2, 27.4]	0.684	0.223
LDH (U/L)	298.0 [257.8, 347.0]	326.5 [284.0, 388.8]	<0.001	285.5 [242.8, 318.8]	315.0 [275.2, 393.5]	0.005	0.152
SII	594.1 [412.8, 862.6]	709.1 [424.1, 1246.8]	0.017	567.9 [378.4, 901.8]	669.2 [386.8, 1246.8]	0.524	0.610
Sex, *n* (%)			0.330			0.403	0.914
Female	135 (52.33)	78 (46.99)		34 (53.12)	18 (42.86)		
Male	123 (47.67)	88 (53.01)		30 (46.88)	24 (57.14)		

Data are presented as median [first quartile, third quartile] or *n* (%). Continuous variables were compared using the Mann–Whitney U test, and categorical variables were compared using the *χ*^2^ test with Yates’ continuity correction. Within-cohort *P* values represent comparisons between the NSMPP and SMPP groups within the training or testing cohort, whereas the between-cohort *P* values represent comparisons between the overall training and testing cohorts. All tests were two-sided, and *P* < 0.05 was considered statistically significant. SII was calculated as platelet count ×  neutrophil count/lymphocyte count. IQR, interquartile range; NSMPP, non-severe *Mycoplasma pneumoniae* pneumonia; SMPP, severe *Mycoplasma pneumoniae* pneumonia; WBC, white blood cell count; NEUT, neutrophil; LYMPH, lymphocyte; MONO, monocyte; PLT, platelet count; CRP, C-reactive protein; PCT, procalcitonin; ALT, alanine aminotransferase; AST, aspartate aminotransferase; CK, creatine kinase; CK-MB, creatine kinase-MB isoenzyme; LDH, lactate dehydrogenase; SII, systemic immune-inflammation index.

### Variable selection

#### Clinical feature selection

Clinical variables were first compared between the NSMPP and SMPP groups in the training cohort using the independent-samples t-test, Mann–Whitney U test, chi-square test, or Fisher's exact test, as appropriate. Variables with *P* < 0.05 in the preliminary group comparison were retained for further analysis. Subsequently, univariable logistic regression analysis was performed on the retained clinical variables to evaluate their associations with SMPP. Given that several hematological and inflammatory indicators were biologically or mathematically correlated, candidate variables were further assessed for multicollinearity using Spearman correlation analysis and variance inflation factor. After removing highly correlated variables or variables with obvious multicollinearity, the remaining low-collinearity features were entered into multivariable logistic regression analysis. Finally, LDH, CK-MB and absolute lymphocyte count remained statistically significant and were selected as the final clinical predictors for clinical machine learning model construction and subsequent integration with the radiomics signature. (Table 2, which only lists variables with statistical significance in the multivariate analysis).

**Table 2:** Results of multivariable logistic regression analysis.coef, coefficient; OR, odds ratio; CI, confidence interval; CK-MB, creatine kinase-MB; LDH, lactate dehydrogenase; LYMPH_value, absolute lymphocyte count.

Feature	Coef	OR	95% CI	P
LDH	0.6449	1.906	(1.459, 2.489)	<0.001
CK-MB	−0.4727	0.623	(0.482, 0.806)	<0.001
LYMPH_value	−0.4255	0.653	(0.516, 0.828)	<0.001

Pairwise correlation analysis was conducted to assess potential collinearity among CK-MB, LDH, and absolute lymphocyte count in the training and testing cohorts. CK-MB and LDH demonstrated only weak-to-moderate positive correlations in both cohorts, while absolute lymphocyte count showed weak or negligible correlations with CK-MB and LDH (Figure 3). No strong pairwise correlation was observed among the three variables. Combined with the variance inflation factor results, these findings indicated no substantial multicollinearity among the selected clinical predictors. Accordingly, CK-MB, LDH, and absolute lymphocyte count were retained for subsequent model development.

**Figure 3:**
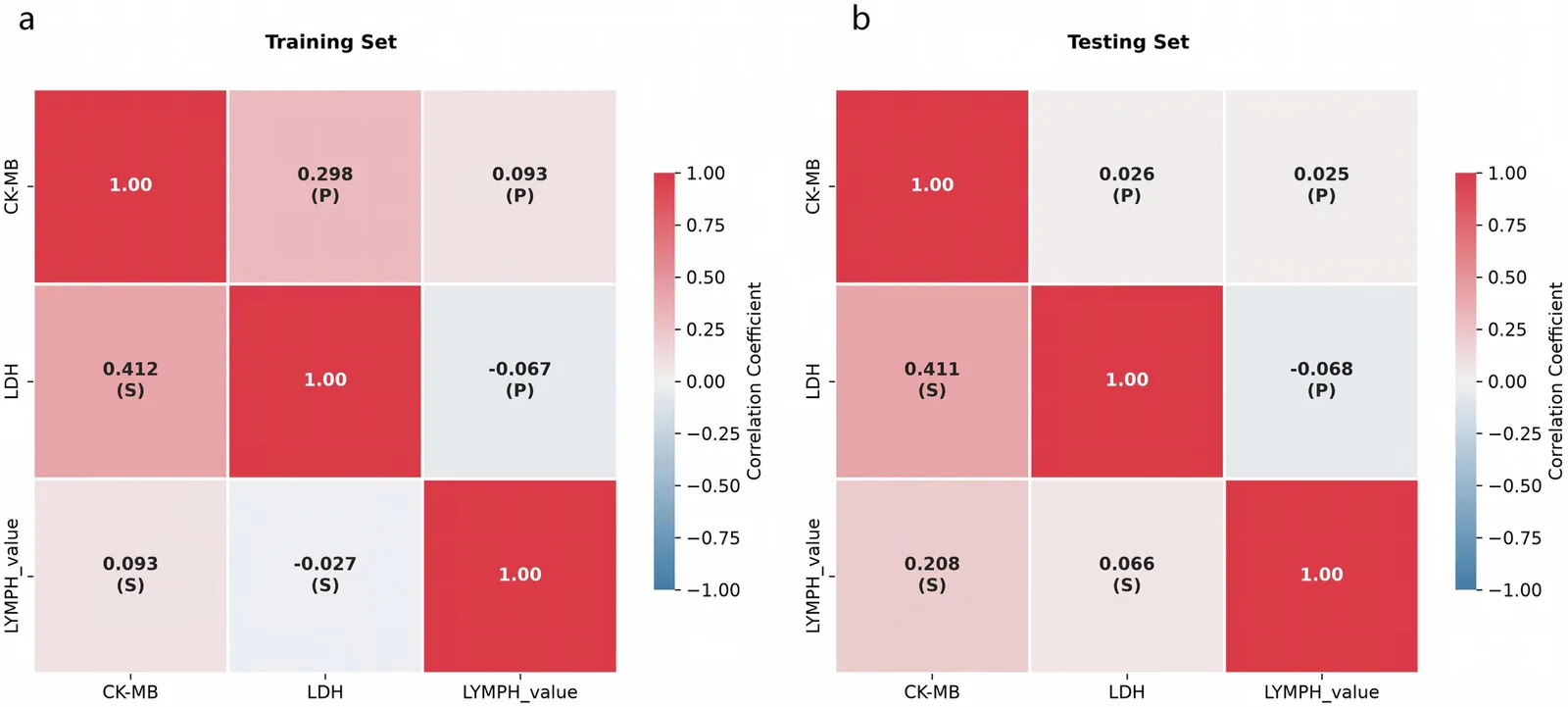
Heat map analysis of correlations between 3 variables. **(a)** Training cohort. **(b)** Test cohort. CK-MB, creatine kinase-MB; LDH, lactate dehydrogenase, LYMPH_value, absolute lymphocyte count.

As shown in Figure 4 and Table 3, in the training cohort, the Clinical model, Radiomics model, and Integrated model achieved AUCs of 0.785 (95% CI: 0.740–0.826), 0.794 (95% CI: 0.748–0.836), and 0.823 (95% CI: 0.780–0.863), respectively. The Integrated model showed the highest accuracy (0.726) and F1 score (0.676), whereas the Radiomics model demonstrated the highest sensitivity (0.735). In the testing cohort, the corresponding AUCs were 0.747 (95% CI: 0.646–0.837), 0.787 (95% CI: 0.683–0.877), and 0.801 (95% CI: 0.699–0.889), respectively. The Integrated model also achieved the highest accuracy (0.745), F1 score (0.697), precision (0.660), and specificity (0.750) in the testing cohort. However, its sensitivity was identical to that of the Radiomics model (0.738), and the absolute improvement in AUC over the Radiomics model was limited to 0.014.

**Figure 4:**
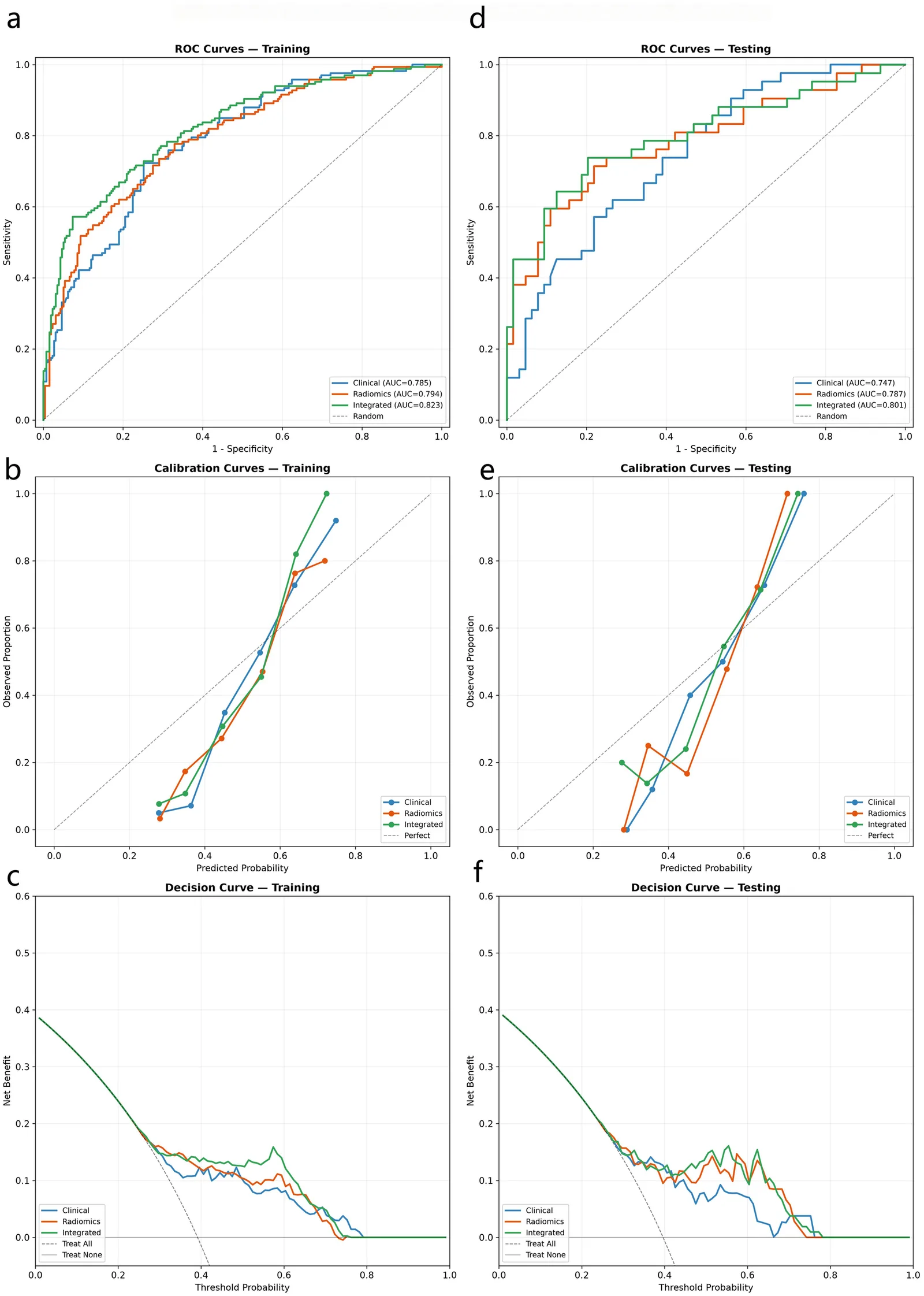
Predictive performance of clinical, radiomics, and integrated models across training and testing cohorts for severity stratification. **(a,d)** ROC curves, **(b,e)** Calibration curves, **(c,f)** Decision curves for training (left), and testing (right) cohorts. AUC, area under the receiver operating curve.

**Table 3:** Diagnostic performance of different models. AUC = Area Under the Curve; CI = Confidence Interval.

Model	AUC (95% CI)	Accuracy	F1Score	Precision	Sensitivity	Specificity
Training cohort						
Clinical	0.785 (0.740–0.826)	0.717	0.634	0.642	0.627	0.775
Radiomics	0.794 (0.748–0.836)	0.717	0.670	0.616	0.735	0.705
Integrated	0.823 (0.780–0.863)	0.726	0.676	0.630	0.729	0.725
Testing cohort						
Clinical	0.747 (0.646–0.837)	0.689	0.582	0.622	0.548	0.781
Radiomics	0.787 (0.683–0.877)	0.736	0.689	0.646	0.738	0.734
Integrated	0.801 (0.699–0.889)	0.745	0.697	0.660	0.738	0.750

Pairwise comparisons further characterized the incremental value of combining clinical and radiomics features. In the training cohort, the Integrated model significantly outperformed the Radiomics model according to both the DeLong test (AUC difference = 0.0295, *P* < 0.001) and IDI analysis (IDI = 0.0099, *P* = 0.0057). The Integrated model also improved discrimination over the Clinical model according to IDI analysis (IDI = 0.0272, *P* = 0.0144), although their AUC difference was not statistically significant in the DeLong test (*P* = 0.1036). In the testing cohort, the Integrated model showed a significant IDI improvement over the Clinical model (IDI = 0.0389, *P* = 0.0466), but the AUC difference remained nonsignificant (*P* = 0.2531). Importantly, neither the DeLong test (*P* = 0.2461) nor IDI analysis (IDI = 0.0066, *P* = 0.2878) demonstrated a significant improvement of the Integrated model over the Radiomics model in the testing cohort. (Table 4).

**Table 4:** Pairwise comparisons of the discriminatory performance of the clinical, radiomics, and integrated models in the training and testing cohorts.

Cohort	Comparison	AUC		DeLong test		IDI analysis		
		AUC 1	AUC 2	Z value	*P* value	IDI	Z value	*P* value
Training cohort								
	Clinical vs. Radiomics	0.7845	0.7937	−0.326	0.7446	0.0173	1.258	0.2082
	Clinical vs. Integrated	0.7845	0.8232	−1.628	0.1036	0.0272	2.448	0.0144
	Radiomics vs. Integrated	0.7937	0.8232	−4.182	<0.001	0.0099	2.766	0.0057
Testing cohort								
	Clinical vs. Radiomics	0.7470	0.7868	−0.746	0.4558	0.0322	1.376	0.1689
	Clinical vs. Integrated	0.7470	0.8010	−1.143	0.2531	0.0389	1.990	0.0466
	Radiomics vs. Integrated	0.7868	0.8010	−1.160	0.2461	0.0066	1.063	0.2878

AUC1 and AUC2 represent the AUCs of the first and second models listed in the “Comparison” column, respectively. The DeLong test was used to compare the AUCs of the two models. IDI indicates the improvement in discrimination achieved by the second model relative to the first model; a positive IDI value favors the second model. A two-sided *P* value < 0.05 was considered statistically significant. The Clinical model was constructed using clinical variables, the Radiomics model using radiomics features, and the Integrated model using both clinical variables and radiomics features.

SHAP analysis demonstrated broadly similar feature contribution patterns in the training and testing cohorts (Figure 5). *logarithm_firstorder_Median* was the most influential predictor in both cohorts. Higher values of *logarithm_firstorder_Median* and LDH generally shifted model predictions toward SMPP, whereas higher lymphocyte counts and CK-MB levels shifted predictions toward NSMPP. The radiomics features *wavelet_LLL_ngtdm_Strength*, *wavelet_HLL_glcm_Correlation*, and *original_shape_Elongation* also contributed to the predictions, although their effects were comparatively smaller.

**Figure 5:**
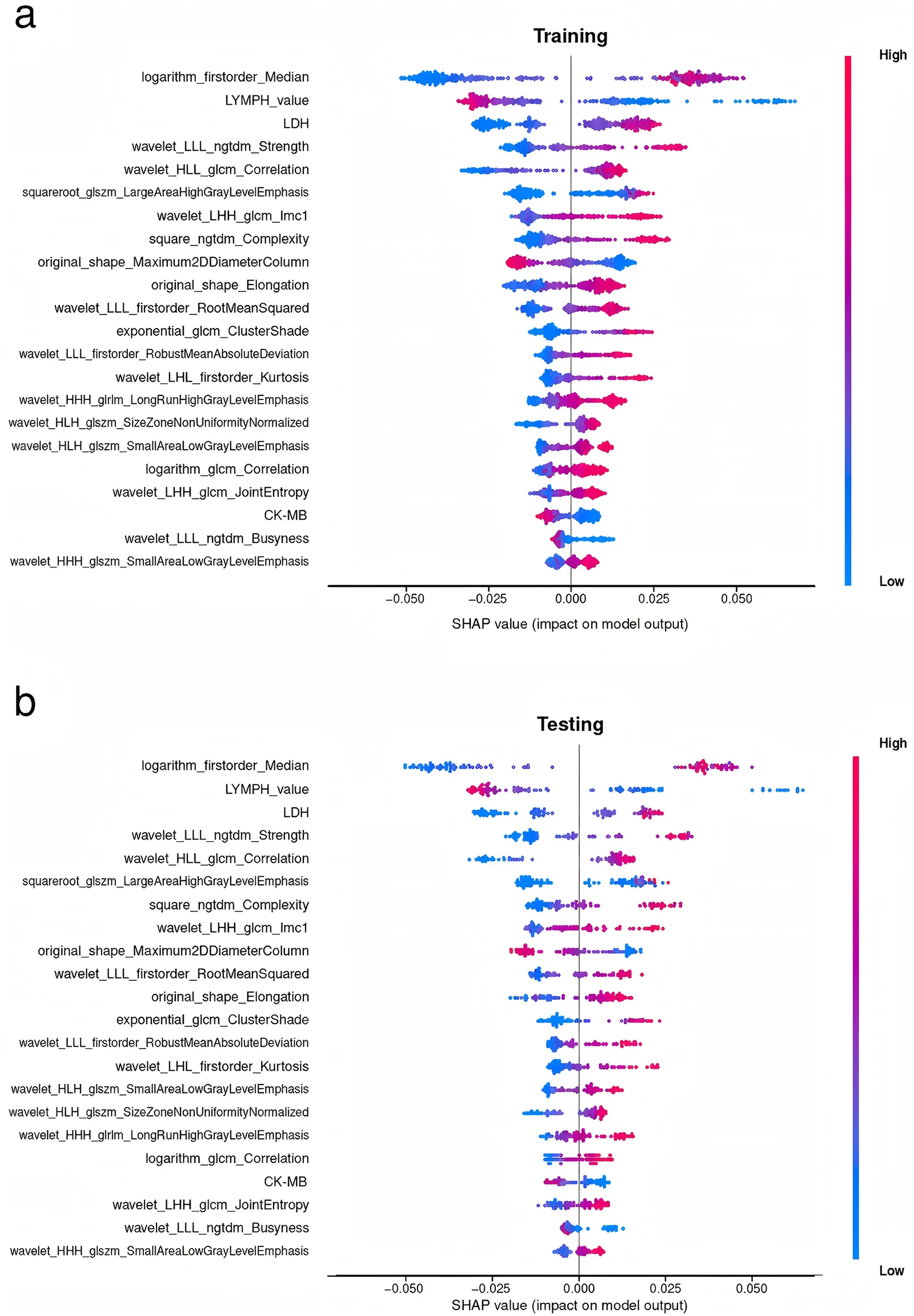
SHAP summary plots for the integrated model. **(a)** Training cohort; **(b)** testing cohort. Features are ranked from top to bottom according to their overall contribution to the model output. Each dot represents one patient. The horizontal position indicates the SHAP value: positive values increase the model output toward severe *Mycoplasma pneumoniae* pneumonia (SMPP), whereas negative values decrease the model output. Dot color represents the original feature value, ranging from low (blue) to high (red). SHAP, Shapley additive explanations; LYMPH, absolute lymphocyte count; LDH, lactate dehydrogenase; CK-MB, creatine kinase–myocardial band; GLCM, gray-level co-occurrence matrix; GLSZM, gray-level size-zone matrix; GLRLM, gray-level run-length matrix; NGTDM, neighboring gray-tone difference matrix; IMC1, informational measure of correlation 1; 2D, two-dimensional. In the wavelet feature names, L and H denote low-pass and high-pass filters, respectively.

## Discussion

In the present study, extrapulmonary manifestations were more frequent in patients with SMPP than in those with NSMPP, suggesting greater systemic involvement in severe disease. However, this finding should be interpreted cautiously because extrapulmonary organ complications were among the criteria used to define SMPP; therefore, the observed difference may partly reflect the outcome definition. Consistent with the clinical heterogeneity of extrapulmonary involvement, Matea et al. described diverse mucocutaneous manifestations of *M. pneumoniae* infection in children (18).

Lung ultrasound radiomics showed potential for predicting SMPP in children. In this single-center study with internal validation, the Radiomics model demonstrated more stable performance across the training and held-out testing cohorts than the Clinical model, whereas the Integrated model achieved the numerically highest AUC in the held-out testing cohort. However, its improvement over the Radiomics model was modest and not statistically significant. SHAP analysis indicated that clinical biomarkers reflecting tissue injury, myocardial involvement, and immune status—including LDH, CK-MB, and absolute lymphocyte count—as well as radiomics features characterizing lesion morphology and texture heterogeneity contributed to model predictions. These findings suggest that clinical indicators may provide complementary information to lung ultrasound radiomics for noninvasive severity assessment, although a definite incremental benefit was not established. Because no external validation was performed, future multicenter studies using independently recruited external cohorts are needed to assess the generalizability and clinical utility of these models.

In the present study, multivariable logistic regression identified LDH as an independent risk factor for SMPP, whereas absolute lymphocyte count and CK-MB were independently associated with a reduced risk of SMPP. Specifically, higher LDH levels were associated with increased odds of SMPP, while higher absolute lymphocyte count and CK-MB were associated with lower odds after adjustment for other clinical variables. This finding is biologically plausible for LDH, as LDH is a marker of tissue injury and inflammatory burden, and previous studies have shown that elevated LDH is associated with refractory, necrotizing, or severe *Mycoplasma pneumoniae* pneumonia in children (19–23). The protective association of Absolute lymphocyte count may reflect the role of preserved lymphocyte-mediated immune regulation during infection; conversely, lymphocyte depletion or an increased neutrophil-to-lymphocyte ratio has been linked to poor outcomes in MPP (24–26). CK-MB is generally considered a marker of myocardial injury in children with MPP or critical illness, and its protective direction in our multivariable model should therefore be interpreted cautiously as an adjusted statistical association rather than evidence that CK-MB itself prevents disease progression (27, 28). Importantly, SHAP analysis provided model-level interpretability consistent with these regression findings: higher LDH values pushed the Integrated model toward higher predicted SMPP risk, whereas higher absolute lymphocyte count and CK-MB contributed to lower predicted risk. Because SHAP explains feature contributions to model output rather than causal effects (29), these findings suggest that LDH, absolute lymphocyte count, and CK-MB are clinically meaningful predictors within the Integrated model, supporting the complementary value of laboratory biomarkers and ultrasound radiomics for early SMPP risk stratification.

SHAP analysis provided a clinically accessible interpretation of the information used by the Integrated model. Across both the training and testing cohorts, model predictions were driven primarily by four aspects of pulmonary consolidation: the overall distribution of echo intensity, the coarseness and spatial organization of internal echoes, lesion shape, and clinical indicators of systemic inflammation or tissue injury. Higher values of logarithm_firstorder_Median, wavelet_LLL_ngtdm_Strength, and wavelet_HLL_glcm_Correlation were predominantly associated with positive SHAP values and therefore with a higher model-predicted probability of SMPP. In ordinary clinical terms, the model identified differences in the typical echo intensity of the lesion and in whether its internal echoes formed relatively coarse or spatially organized patterns. These quantitative characteristics may capture subtle differences within pulmonary consolidation that are difficult to assess consistently by visual inspection alone. Radiomics is designed to convert medical images into quantitative descriptors of intensity, texture, and morphology, thereby potentially revealing imaging phenotypes beyond subjective visual assessment (30–32).

These features also have biologically plausible, although indirect, interpretations. Normal aerated lung largely prevents ultrasound transmission, whereas pulmonary consolidation becomes visible when alveolar air is reduced or replaced by inflammatory cells, edema, or exudative material (33). In severe MPP, an excessive host inflammatory response may produce more extensive tissue injury and may be accompanied by dense consolidation, mucus plugging, residual aerated bronchi, atelectatic change, and, in some patients, necrotizing injury (3, 34). The altered tissue–air–fluid composition may therefore contribute to changes in the transformed grayscale distribution represented by logarithm_firstorder_Median. Similarly, NGTDM Strength and GLCM Correlation may indirectly capture the coexistence and spatial arrangement of consolidated tissue, residual air, air bronchograms, edema, and inflammatory exudate, resulting in differences in echo coarseness and internal organization. The positive SHAP contributions of higher NGTDM Strength and GLCM Correlation suggest that more pronounced coarse-scale echo patterns and stronger spatial dependency between neighboring grayscale values contributed to the model's classification of SMPP. Nevertheless, because these features were calculated after logarithmic or wavelet transformation, they should not be interpreted simply as lesions being “brighter” or “more heterogeneous” on routine visual assessment.

Lesion morphology provided additional predictive information. In PyRadiomics, original_shape_Elongation is defined as the ratio between the minor and major principal axes; a value closer to 1 represents a more rounded or less elongated lesion, whereas a value closer to 0 represents a more elongated configuration (31). In the present SHAP plots, higher Elongation values were generally associated with a higher predicted probability of SMPP. Thus, the model tended to associate a relatively less elongated lesion outline on the selected ultrasound image with severe disease. This may reflect differences in the anatomical distribution or extension pattern of consolidation. However, this interpretation should remain cautious because the apparent shape of a pulmonary lesion depends on the scanning plane, the selected two-dimensional image, lesion contact with the pleural surface, and manual ROI delineation. It should not be interpreted as evidence that more rounded lesions are intrinsically more severe.

The contribution of quantitative imaging features is broadly consistent with a recent multicenter CT-based radiomics study in MPP, which demonstrated that radiomics features could provide information for predicting disease severity and delayed recovery (35). However, CT and ultrasound represent different physical imaging processes, and direct biological equivalence between their radiomics features cannot be assumed. More importantly, the present SHAP results explain how individual variables influenced model output but do not demonstrate biological causality. Ultrasound radiomics features are also affected by imaging depth, gain, transducer frequency, reconstruction and post-processing algorithms, device vendor, and ROI segmentation. Because histopathological specimens and direct image–pathology correlation were unavailable, the selected features should be regarded as quantitative imaging phenotypes and hypothesis-generating markers rather than specific histopathological biomarkers. Prospective multicenter studies using harmonized acquisition protocols and, where feasible, correlation with bronchoscopy, laboratory markers, or pathological findings are needed to validate their biological significance.

Notably, the Radiomics model showed stable performance in the testing cohort, suggesting that lung ultrasound radiomics features may provide robust and reproducible information for predicting severe MPP in children. Although the integrated model achieved the highest AUC, its incremental improvement over the Radiomics model was limited and was not statistically significant by DeLong testing. This indicates that radiomics features may represent the major predictive component of the integrated model, while clinical biomarkers provide additional complementary information. SHAP analysis further demonstrated that features reflecting gray-level intensity, lesion morphology, and texture heterogeneity were important contributors to model prediction, together with clinical biomarkers such as LDH, CK-MB and absolute lymphocyte count. These findings support the concept that ultrasound radiomics can capture subtle lesion heterogeneity beyond conventional visual assessment and may serve as a quantitative imaging biomarker for noninvasive severity assessment in pediatric MPP.

### Limitations

First, the retrospective study design may have introduced selection bias and resulted in optimistic estimates of model performance. A substantial proportion of potentially eligible patients were excluded because lung ultrasound examinations were unavailable or were not performed within the predefined time window. Because ultrasound examinations were obtained as part of routine clinical practice rather than through systematic prospective screening, the included patients may have differed from the excluded population in disease severity, clinical management, or access to imaging. Consequently, the study population may not fully represent the broader population of children with MPP. Second, the absence of longitudinal ultrasound and laboratory data limited our ability to assess temporal changes during disease progression and recovery, including treatment response, macrolide unresponsiveness, delayed radiological resolution, and residual pulmonary abnormalities. Third, lung ultrasound images were acquired using different ultrasound systems. Variations in acquisition parameters, spatial resolution, grayscale mapping, and vendor-specific post-processing algorithms may have affected radiomics feature values and model performance. Although a relatively standardized image acquisition and analysis protocol was applied, device-related heterogeneity could not be completely eliminated. Fourth, both the training and testing cohorts were derived from the same institution; therefore, the testing cohort represented internal rather than true external validation, potentially limiting the generalizability of the findings to other institutions, patient populations, and ultrasound systems. Moreover, disease severity was defined according to existing pediatric clinical criteria, which may not fully capture the biological and clinical heterogeneity of severe MPP. Future prospective multicenter studies should employ systematic patient enrollment, harmonized ultrasound acquisition protocols, longitudinal follow-up, and detailed treatment records. Feature robustness assessment, batch-effect correction, and independent external validation should also be performed to improve the reproducibility, cross-device generalizability, and clinical applicability of the model.

## Conclusion

Lung ultrasound radiomics demonstrated stable discriminatory performance for identifying severe *Mycoplasma pneumoniae* pneumonia in children and provided quantitative information complementary to conventional clinical indicators. The Integrated model achieved the highest overall AUC, although its incremental improvement over the Radiomics model was limited in the testing cohort, indicating that radiomics features represented an important source of predictive information while clinical variables provided additional value. SHAP analysis identified grayscale intensity, lesion morphology, texture heterogeneity, and clinical biomarkers as important contributors to model predictions, supporting the potential of lung ultrasound radiomics as a non-invasive tool for early risk stratification in pediatric MPP.

## Data Availability

The raw data supporting the conclusions of this article will be made available by the authors, without undue reservation.
